# Bringing Light to the Darkness: COVID-19 and Survivance of American Indians and Alaska Natives

**DOI:** 10.1089/heq.2020.0123

**Published:** 2021-02-25

**Authors:** Spero M. Manson, Dedra Buchwald

**Affiliations:** ^1^ Centers for American Indian and Alaska Native Health, Colorado School of Public Health, University of Colorado Anschutz Medical Campus, Aurora, Colorado, USA.; ^2^ Institute for Research and Education to Advance Community Health, Washington State University, Seattle, Washington, USA.

**Keywords:** American Indians, Alaska Natives, COVID-19, risk, survivance

## Abstract

The novel severe acute respiratory syndrome coronavirus 2 (SARS-CoV-2) sweeping across our country has reawakened the fear, pain, stigma, and loss of past outbreaks of infectious diseases among American Indians and Alaska Natives. Attention to the pandemic has emphasized the challenges it poses for Native peoples: their vulnerability, the heartbreaking battle to constrain contagion, the lack of resources to care for those afflicted by the virus, and the mounting consequences for individuals, families, and community. We highlight the factors that contribute to them but conclude by underscoring the intrinsic strengths and resilience, which, in combination with modern public health tools, promise to resolve them.

## Introduction

“Once more darkness descends upon our people and this land. It bears many names: smallpox, the flu, measles, typhoid, TB. Today … a virus. Suffering and death always follow. Yet our traditions shine brightly, casting light to guide the way. We again struggle to survive, buoyed by these traditions, now armed as well with medicine and science, weapons of the Western world. The challenge is how to bring both to bear on this danger so we may live on.” An Athabascan Elder

[translated]

As these powerful words of an Athabascan elder underscore, the novel severe acute respiratory syndrome coronavirus 2 (SARS-CoV-2) sweeping across our country has reawakened the fear, pain, stigma, and loss of past outbreaks of infectious diseases among American Indians and Alaska Natives. It amplifies fractures in the social fabric only recently covered over and exacts a terrible toll by exacerbating the health disparities that place this special population at added risk of sickness and death.

The public has had brief glimpses into these circumstances, largely through the eyes of the Diné (Navajo Nation) in the southwest and the Lakota of the northern plains. Most of this attention emphasized challenges, namely, their vulnerability, the heartbreaking battle to constrain contagion, the lack of resources to care for those afflicted by the virus, and the mounting consequences for individuals, families, and community. Our remarks highlight the factors that contribute to these challenges and to their possible resolution. We then turn to the historical context that adds salience to the current pandemic and how the attendant memories of past infectious outbreaks resonate even more loudly among Native peoples than may be supposed by those unfamiliar with their history. The added layer of past experiences acts as a lens through which to view problem and solution, action and inaction, as well as responsibility and obligation. Our discussion closes by emphasizing the strengths again evident as tribal communities address these challenges. It is not simply a matter of survival, but of survivance: recapitulating a way of life that nourishes indigenous ways of knowing, this time extended by lessons from a contemporary pandemic.^1^

### The landscape

SARS-CoV-2 has deeply penetrated tribal communities. As of mid-May 2020, American Indians comprised 18% of COVID-19-related deaths and 11% of SARSCoV-2 cases compared with 4% of the total population in Arizona, 57% of cases compared with 9% of the total population in New Mexico, and 30% of cases compared with 2% of the total population in Wyoming.^2^ Within the same timeframe, the Indian Health Service (IHS) reported 5500 positive cases at IHS, tribal, and urban Indian facilities. The infection rate among the Diné surpassed the state of New York, the center of the U.S. pandemic: 2680 cases per 100,000 people compared with 1890. Today, the Navajo Nation has the highest per capita incidence of COVID-19 infection in the United States despite draconian control measures.^3,4^ The situation is even worse among smaller tribes. In the Pueblo of Zia, population 934 people, the rate was 3319 per 100,000—10 times the rate observed in the general population of New Mexico, and almost double the rate in New Jersey, the second hardest hit among all U.S. states. The Pueblo of San Felipe, population 3544, is also badly affected with 3301 known cases per 100,000.^5^ These reports likely underestimate the actual number of cases due to restricted testing capacity and a lag in reporting.

As of this writing, 1 month later, IHS projections nearly doubled to 9223 total cases and 365 deaths.^5,6^ American Indians and Alaska Natives are disproportionately represented not only among cases but also among those who have died from COVID-19. Native residents of New Mexico, for example, account for nearly 60% of coronavirus deaths but constitute just 8.8% of the population.

SARS-CoV-2 presents elevated health risks for Native peoples who experience substantial health disparities.^7,8^ They suffer disproportionately high prevalence of many health conditions that place them at greater risk for serious illness and death if they contract coronavirus, including diabetes, heart disease, asthma, and obesity.^9–12^ The Centers for Disease Control and Prevention finds that 34% of American Indians and Alaska Natives <65 years are at risk of serious disease from SARS-CoV-2 compared with 21% of nonelderly white adults.^13^ Living conditions also put Native people at increased risk for exposure to the disease. For example, compared with other groups, American Indians and Alaska Natives are more likely to lack access to clean water and plumbing^14^ and to live in substandard and crowded housing,^15,16^ thereby limiting their ability to practice frequent handwashing and social distancing.^17^

### Barriers to care

American Indians and Alaska Natives face barriers accessing health care that also hinder obtaining coronavirus testing and treatment services. For example, compared with their white counterparts, Native people <65 years are more likely to have not seen a doctor in the past year due to cost (19% vs. 13%) and to have delayed care for other reasons (36% vs. 19%).^8^ IHS is the primary vehicle through which the federal government fulfills its responsibility to provide primary health care services to this special population. However, IHS has been chronically underfunded to meet the health care needs of Native people.^18^ Across the country, 25% of health care provider positions were vacant, with 30% vacant within the Navajo Nation as of 2018.^19^ The IHS budget only provides $4078 per capita for health care spending—less than half of what is spent for federal beneficiaries in the general population—and covers just 16% of the estimated funding needed to fully fund all IHS federally operated, tribally operated, and urban Indian-operated facilities.^18,20^ IHS clinics are located predominantly on or near reservations in rural areas, making them inaccessible to urban American Indians and Alaska Natives, who represent >70% of the population.^21^

Urban American Indians and Alaska Natives are served by 41 Urban Indian Health Programs located in cities with large numbers of Native residents.^22^ Although these facilities provide a range of social, behavioral, and medical services, they too are dramatically underfunded. This complex and fragmented system hinders the ability of Native people to obtain high-quality health care.^17^ Perhaps surprisingly, as of 2018, 22% of American Indians and Alaska Natives <65 years were uninsured, the highest of all racial and ethnic groups.^23^

Tribes support a wide array of basic governmental services traditionally funded by state or local government. Absent traditional tax bases, for the past 30 years, the economic health of tribes has been enhanced by gaming and nongaming business enterprises, with the former contributing an increasing majority of their revenue. The SARS-CoV-2 crisis has had a devastating effect on both sectors. To protect their members and communities, tribes closed >500 casinos and most nongaming businesses, which had produced >1.1 million jobs and >$49.5 billion in annual wages and benefits in 2019.^24^ Like non-Native businesses across the country, massive layoffs ensued; workers lost insurance coverage; and their savings have been drained to survive. The accruing debt among an already impoverished population is especially crippling.

Imagine, then, the impact of the promised, but delayed distribution of $8 billion in funds earmarked for tribes in the Coronavirus Aid, Relief, and Economic Security (CARES) Act. In the minds of many Native people, the CARES Act constituted yet another example of poor treatment at the hands of the federal government. Moreover, incautious language in the Act led to a legal dispute between federally recognized tribes and Alaska's tribal corporations as to who is entitled to the aid, rupturing deep political wounds among tribal entities that had begun to heal in the past 25 years.

### Pandemics: past and present

As the Athabaskan elder's remarks earlier remind us, American Indian and Alaska Natives are no strangers to epidemics. The current novel SARS-CoV-2 crisis is one in a long history of deadly viruses to plague Native peoples. European arrival in the late 1400s and beyond introduced smallpox, bubonic plague, chickenpox, measles, diphtheria, influenza, malaria, scarlet fever, typhoid, tuberculosis, and pertussis: diseases to which Native people bore no natural immunity.^25–27^ The consequences were devastating, killing an estimated 90% of this population, with lingering effects such as social and cultural dislocation, historical trauma, and population migrations well into the 1960s.^28^

The smallpox epidemic of the 1830s is generally regarded as the deadliest of plagues to afflict Alaska's Native population. Ravaging communities across the state, the epidemic wiped out entire villages, killing upward of two-thirds of Alaska Native people in the lower Yukon area alone.^29^ During the Spanish influenza pandemic of 1918, >80% of influenza deaths were among Native people, who comprised 48% of the total state population.^30^ Likewise, the mortality rate of the disease was estimated to be four times higher among American Indians than for whites.^31^ Similar accounts can be readily found with respect to tuberculosis, which by the end of the 19th century had become a deadly health problem. American Indians accounted for 45 per 1000 total deaths in Nevada and up to 625 per 1000 total deaths in New York: manyfold greater than their non-Indian counterparts. Regardless of the infectious disease, history repeatedly has documented that Native people were highly vulnerable, suffered markedly from the consequences of contagion, and died in excess than other segments of American society. But the consequences cannot be measured simply in terms of mortality alone.

Infectious disease-related mortality contributed to enormous social and cultural upheaval among American Indians and Alaska Natives.^28,32^ Entire families, even tribal communities, were decimated; many survivors experienced major lasting disruptions. As was common among the poor, regardless of race, newspaper articles and personal stories about the Spanish influenza speak to Alaska Native and American Indian children relocated *en masse* to institutional settings distant from their homelands to which many never returned.^33^ The same media sources describe the advent of sanitariums to address tuberculosis in the 1920s and 1930s. Fueled by government-funded campaigns, Native people were often sent to Seattle, WA; Lapwai, ID; Rapid City, SD; Phoenix, AZ; and other locations where they spent years housed in converted public health hospitals, vacated boarding schools, and former jails awaiting a cure. Families dissolved, parenting skipped generations, cultural practices languished, and personhood and collective identity came under major assault.

Not unlike today, “blame the victim” beliefs paraded as explanations for the vulnerability of American Indians and Alaska Natives to the ravages of these pandemics, and to their difficulty weathering the consequences. Unsanitary living conditions, poor personal hygiene, communal life, and ignorance were universal risks, regardless of race/ethnicity. However, observers of the day specifically emphasized these circumstances in their stories about why contagion flourished in tribal communities. From the late 1800s to early 20th century, “dirty” and “illiterate” regularly appeared in newspaper accounts of the spread of smallpox, tuberculosis, and influenza among Native peoples. Before dismissing these attributions as belonging to a distant past, one only need cast back to the 1993 reports of the Hantavirus in the Southwest United States, initially known as the “Navajo plague,” “Four Corners virus,” and the Centers for Disease Control and Prevention (CDC)'s label “Muerto Canyon Hantavirus.” Mouse droppings associated with its transmission were linked to descriptions of reservation dwellings as “filthy,” “squalid,” and “unhygienic.” Stigma abounds, now and then.

This unfortunate history deeply colors American Indian and Alaska Native views of the SARS-CoV-2 crisis. They fear the social and cultural disruptions that history has shown to be close companions of outbreaks, epidemics, and pandemics. Seldom the masters of their own fate, often subject to external forces imposed upon them by the federal government, Native people are suspicious of promises of aid that have often proved to be hollow offerings. As a result, tribal communities today emphasize self-governance and tribal sovereignty, and seek greater control over their lives, lands, and health care systems. During the current pandemic, the most vivid examples include the Diné and Cheyenne River Sioux blockades of roads leading into their respective reservation lands, and special application procedures required for admission.^34,35^ The differential response of the states that encompass these tribal lands illustrates new alliances and old enmities. The governor of New Mexico strongly supported the Diné in their efforts at mitigation, working closely with the tribe to the benefit of both. In sharp contrast, the governor of South Dakota vehemently opposed Sioux measures to limit spread of SARS-CoV-2, threatening legal and even forcible intervention.^36^ In Alaska, with full support of local government, some Native villages ended air flights into their communities in attempts to self-isolate and are only now cautiously opening up with borough and state assistance.

The survivance of Native peoples is evident in other ways as well. Traditional healing practices have been mobilized to emphasize indigenous medicine plants to address certain symptoms, boost the immunological system, and emphasize self-care.^37^ Other traditions have been adapted to increase a sense of personal- and group-efficacy,^38–40^ yielding fresh approaches to social support, such as Diné youth caring for elders^41^ or a Native-owned Minneapolis restaurant providing meals of rabbit stew, venison, walleye, and wild rice to homebound American Indian elders. Biomedical practitioners have joined with traditional healers to demonstrate how current forms of mitigation, such as handwashing, social distancing, and wearing masks, can enable the latter to safely practice their healing rituals.^42^

## Conclusion

Scholars, health care professionals, advocates, policymakers, and funders have a unique collective opportunity and obligation to join Native peoples in bringing to bear the strengths of tribal communities, of science, and of past and present-day lessons from public health to battle the SARS-CoV-2 pandemic. Not only an opportunity but also a moral imperative, we need to recognize the broader world of which all Americans are a part and to which we can contribute. This is a challenge we are capable of meeting.

## Abbreviations Used

CARES: Coronavirus Aid, Relief, and Economic Security
CDC: Centers for Disease Control and Prevention
IHS: Indian Health Service
SARS-CoV-2: severe acute respiratory syndrome coronavirus 2
